# Communications Biology turns five!

**DOI:** 10.1038/s42003-023-04467-0

**Published:** 2023-01-23

## Abstract

We reflect on how far we have come while looking forward to the next phase of our journey.

C*ommunications Biology* published its first paper in January 2018. The journal was launched to provide a new open access option for research in all fields of biology, offering an alternative venue for findings of high interest to a specialist audience. We envisioned an inclusive biology journal with high standards for reporting, reproducibility, ethics and transparency.

Now, five years later, we have published more than 4000 Research Articles, and over 60 Review and Perspective Articles. We are immensely grateful to the authors and reviewers who have put their trust in a new and ambitious journal. To highlight some of our very first authors, we have interviewed them about their research and experience of the journal; these Q&As can be found here, here and here.

We celebrate our first five years together with our editorial board, who have been instrumental in shaping *Communications Biology*. Our collaborative editorial model is outlined in more detail here. We feel that this model brings together the best of two worlds: the in-house team of PhD-level full-time editors bring their broad knowledge of their field of expertise and help to ensure consistency and adherence to editorial policies. Sharing our commitment to editorial excellence, our editorial board members (EBMs) not only provide their scientific expertise but also their perspective as representatives of their research communities.

Along with an increase in submissions over these five years, our in-house editorial team has expanded to 11 editors including the Chief Editor and two Deputy Editors. Starting with eight EBMs back in January 2018, the *Communications Biology* editorial board has grown to 133 EBMs, who are at different career stages and spread across no less than 29 countries. In 2022 we introduced Section Lead EBMs, who are experienced external editors who are given additional responsibilities. In this video, some of our Section Leads talk about their work and their thoughts on the journal.

We are aware that fairness and timeliness in editorial decisions is very important to our authors and we strive to be as consistent and efficient as possible, while also being thorough and transparent. You can read more about our metrics and editorial values on our website. To improve the transparency of the peer-review process, since January 2019, authors can choose to have referee reports published alongside their paper. In the first half of 2022, 69% of our authors chose transparent peer review, illustrating that this is a welcome option. Since June 2021, we have also published the names of the handling editors for each paper to acknowledge their contributions.

We aim to keep diversity, equity and inclusion at the forefront of everything we do. And, we are proud of our efforts to acknowledge the diversity of the research community. In our series of Q&As, we initially highlighted early career researchers and more recently we have interviewed various researchers from diverse backgrounds and disciplines about their research and experiences. An ongoing goal is to further diversify our reviewer pool.

Our papers span subfields across all biological sciences from biophysics to ecology. While we are proud to provide a home for such a wide range of excellent research, we also showcase specific research areas through Collections. These can either be retrospective or involve an active call for papers, often headed by one of our EBMs such as the latest one on DNA replication and replication stress.

And, speaking of Collections, to highlight some of the great papers we have published in our first five years, we have put together a retrospective collection. We have gathered the papers that attracted attention from the press and on social media. Among these is the study on the attempts to grow plants in moon soil and one on stinging structures in upside-down jellyfish! The in-house editorial team and our EBMs have also selected some of their favourite papers from the last five years. Although this collection includes our most highly cited paper, SPHIRE-crYOLO is a fast and accurate fully automated particle picker for cryo-EM, publications were chosen in the spirit of celebrating our broad scope rather than according to any specific metrics. We hope you will enjoy the selection!

Five years marks a major milestone for *Communications Biology*, but our journey is just beginning. We will continue to serve our specialist communities as an platform for excellent research and celebrate diversity across the biological sciences. Our ethos as a journal is reflected in the dedication of our editors and board members to high quality and reproducible research, and we remain committed to working with all biologists now and in the future.

You are welcome to get in touch with any feedback on the journal or to discuss *Communications Biology* as a potential venue to publish your research. You can sign up to meet the editors, email an individual editor, or contact us on Twitter @commsbio.

